# In Vitro and In Vivo Anti-Inflammatory Potential of Cannabichromene Isolated from Hemp

**DOI:** 10.3390/plants12233966

**Published:** 2023-11-25

**Authors:** Min Hong, Jong-Hui Kim, Joon-Hee Han, Byeong-Ryeol Ryu, Young-Seok Lim, Jung-Dae Lim, Sang-Hyuck Park, Chang-Hyeug Kim, Soo-Ung Lee, Tae-Hyung Kwon

**Affiliations:** 1 Chuncheon Bioindustry Foundation, Chuncheon 24232, Republic of Korea; fabre_min@cbf.or.kr (M.H.); jonghe5820@cbf.or.kr (J.-H.K.); cbfhjh@cbf.or.kr (J.-H.H.); kchyeu@cbf.or.kr (C.-H.K.); 2 Department of Bio-Health Convergence, Graduate School, Kangwon National University, Chuncheon 24341, Republic of Korea; fbqudfuf0419@kangwon.ac.kr (B.-R.R.); potatoschool@kangwon.ac.kr (Y.-S.L.); ijdae@kangwon.ac.kr (J.-D.L.); 3 Institute of Cannabis Research, Colorado State University—Pueblo, Pueblo, CO 81001, USA; sanghyuck.park@csupueblo.edu

**Keywords:** cannabichromene, cytokines, inflammation, pink pepper, RAW 264.7 cells

## Abstract

Cannabichromene (CBC), a non-psychoactive cannabinoid found in *Cannabis sativa*, has recently been shown to possess several medicinal properties. However, how CBC produces anti-inflammatory effects and the mechanisms of this remain poorly studied. Therefore, we extracted and purified the CBC from the *Cannabis sativa* cv. pink pepper (hemp cultivar). The efficacy of CBC in reducing inflammation in RAW 264.7 macrophages and a λ-carrageenan-induced mouse model was then evaluated. CBC had no cytotoxicity up to a concentration of 20 μM and inhibited nitric oxide production by approximately 50% at a concentration of 20 μM. In addition, CBC treatment significantly inhibited causes of inflammation such as inducible nitric oxide synthase (iNOS), interleukin-1β (IL-1β), IL-6, and tumor necrosis factor-α (TNF-α) at both the mRNA and protein levels. Moreover, CBC suppressed LPS-stimulated inflammation in RAW 264.7 cells by downregulating the nuclear factor kappa B (NF-kB) and mitogen-activated protein kinase pathways (MAPK). Furthermore, our in vivo experiments confirmed that the λ-carrageenan-induced increase in the levels of the cytokines iNOS, IL-1β, and IL-6 was abrogated following treatment with CBC. Therefore, CBC has potential anti-inflammatory effects and may be useful for preventing or treating inflammation.

## 1. Introduction

Inflammation is the immune response of the body to irritants, and various factors can cause cell damage [1,2]. It is a complex response that occurs when immune cells such as macrophages and neutrophils are activated, and it is known to be regulated by cytokines such as nitric oxide (NO) [3]. Macrophages are distributed throughout the human body; these cells produce various inflammatory cytokines such as TNF-α, IL-1, and IL-6 and stimulate NO production during inflammation [4]. Acute inflammation is characterized by vasodilation, fluid exudation, and neutrophil infiltration and is activated and amplified by intracellular and extracellular factors that closely orchestrate the inflammatory process [5]. Although primarily a response of the immune system, severe inflammation can promote systemic inflammatory response syndrome, leading to organ damage, shock, and death [5,6]. Inducible nitric oxide synthase (iNOS) and cyclooxygenase-2 are responsible for the production of nitric oxide (NO) and prostaglandin E_2_, which typically play a vital role in combating bacteria and eradicating tumors. Nevertheless, excessive NO production can lead to conditions such as sepsis or inflammatory diseases, including asthma, rhinitis, and cancer [7,8,9,10]. Infectious and noninfectious substances and cellular damage activate inflammatory cells, usually through the nuclear factor kappa B (NF-kB) and mitogen-activated protein kinase (MAPK) pathways [6]. In addition, the carrageenan-induced mouse model is one of the animal experimental models that can be used for evaluating the efficacy of anti-inflammatory agents, and it has been determined that an increase or decrease in inflammation-related cytokines can be confirmed using this experimental model [11,12].

*Cannabis sativa* is an annual and dioecious plant that produces a wide variety of compounds in the glandular trichomes found in the female flowers. These compounds include cannabinoids, terpenes, and flavonoids. A total of more than 400 substances, called cannabidiols, that can be derived from hemp have been identified to date, including Δ9-tetrahydrocannabinol (Δ9-THC), Δ8-tetrahydrocannabinol (Δ8-THC), cannabichromene (CBC), and cannabidiol (CBD) [13]. Plant cannabinoids have been studied in various ways as food and medicinal materials. Among the plant cannabinoids, CBC was first isolated in 1966 by Gaoni and Mechoulam, who also discovered THC and CBD. CBC is structurally similar to THC and CBD, but differs in its chemical composition, which makes it unique in terms of its potential therapeutic benefits. The scope of CBC research includes a wide range of medical applications, including the management of neuroprotection [14], the inhibition of NO production [15], and the improvement in refractory epileptic encephalopathy (CARE-E) [16]. Although some anti-inflammatory effects of CBC have been described [17,18,19], it is important to acknowledge that not all of the biological effects have been comprehensively defined, emphasizing the need for additional ongoing research and investigation.

For this study, we conducted experiments using “Pink Pepper”, a newly developed Korean hemp cultivar. This specific cannabis variety has been selectively bred and cultivated in the Republic of Korea, primarily for medicinal purposes. Additionally, compared with the other varieties measured by Glivar et al., the flower part of this variety has a relatively high concentration of CBC at approximately 0.158% *w*/*w* [20]. The “Pink Pepper” genetic code has been submitted to GenBank [21]. Additionally, detailed annotations, gene structural information, and functional predictions related to this genome can be located within the Figs-Share database [22].

Consequently, we conducted experiments to address the necessity of cultivating “Pink Pepper” strains with elevated CBC content, thus underscoring the industrial significance of this valuable material. Ultimately, our study elucidated the anti-inflammatory properties of CBC and ascertained its industrial applications.

## 2. Results and Discussion

### 2.1. Cell Viability and Suppression of NO Production in RAW 264.7 Cells in response to Cannabichromene

**Figure 1:**
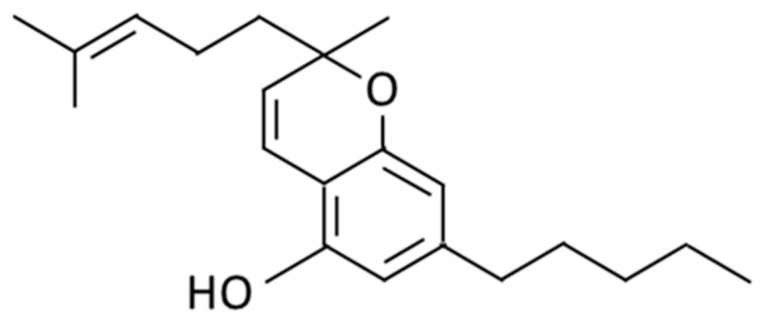
Structure of cannabichromene (CBC).

The structural representation of CBC isolated from “pink pepper” hemp is illustrated in Figure 1. CBC shares structural similarities with CBD, both of which are terpenophenolics consisting of a diphenol and a monoterpene moiety [23]. Structural changes may occur through processes such as carboxylation and halogenation [15]. CBC is a non-psychoactive phytocannabinoid found in cannabis plants [24] that may be responsible for the anti-inflammatory and analgesic effects of cannabis [1].

CBC exhibits both agonistic and antagonistic properties towards a variety of receptors. The effects of cannabinoids are usually mediated by CB1 and CB2 protein-coupled receptors [25]. CB1 receptors usually mediate psychoactive effects and are present in a wide variety of tissues [25]. Notably, CBC exhibits activity across a variety of anatomical sites, including the pancreas, heart, adipose tissue, liver, skeletal muscle, and the reproductive organs [26]. CB2 receptors have been detected in numerous immune cells and a limited number of neurons, and they are recognized as playing a role in inflammation and immune-modulating functions [27]. CBC activates CB2 receptors, but not CB1 receptors [28], and thus is considered a selective CB2 receptor agonist that can be utilized for CB2 receptor-related regulation of inflammation [28]. Furthermore, TRPV receptors and PPARγ receptors represent prominent molecular targets of CBC. There is also the possibility that akin to other cannabinoids, CBC may exhibit an affinity for additional receptors [26,29]. The diverse range of receptor affinities exhibited by CBC underlies its multifaceted pharmacological attributes [26,29], indicating the potential utility of CBC as an economically viable substance capable of eliciting not only anti-inflammatory effects, but also a spectrum of other effects concurrently.

**Figure 2:**
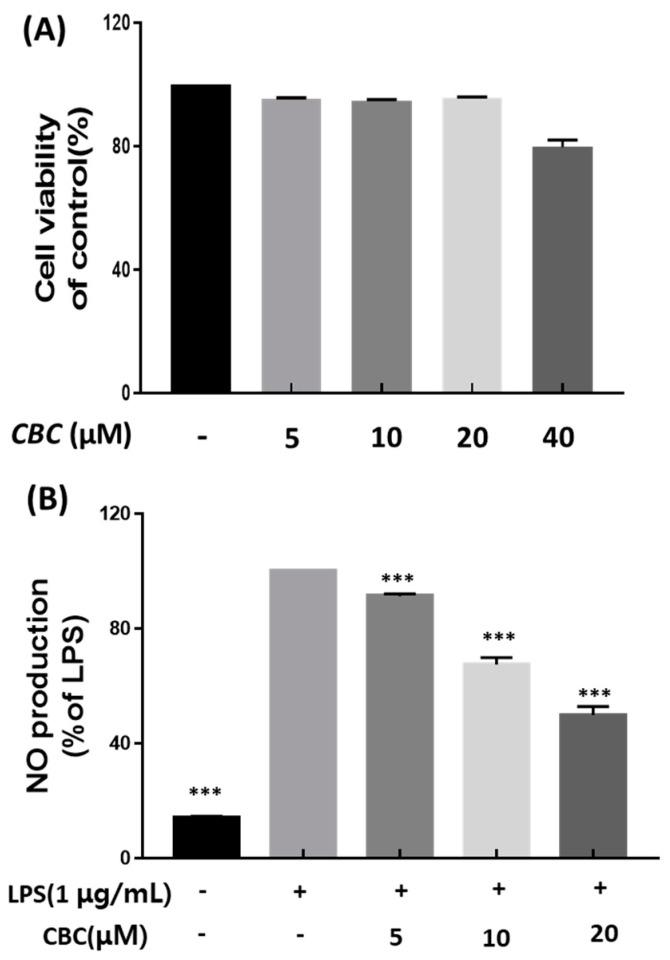
The impact of cannabichromene (CBC) on inflammation in RAW 264.7 cell line-induced LPS. (**A**) Cell viability under varying concentrations of CBC compared with the control group. (**B**) Inhibition of NO production rates at different CBC concentrations in comparison to the LPS-only experimental group. Data is presented as means ± standard deviation for triplicate experiments. *p*-values were evaluated through ANOVA analysis followed by Tukey’s test, *** *p* < 0.001.

The effects of CBC on cell viability were examined using the Cell Counting Kit-8 assay (CCK-8 assay). The results showed that CBC treatment at concentrations of 5, 10, and 20 μM had no effects on RAW 264.7 cell viability compared with the untreated control. However, CBC treatment at a concentration of 40 μM resulted in decreased RAW 264.7 cell viability at 79.66 ± 1.91% compared with the untreated control. These studies confirmed that there was no toxicity to the cells at the maximum treatment concentration of 20 μM, and further experiments were conducted based on the aforementioned treatment concentrations (Figure 2A). Finally, we performed CBC treatment at three different concentrations (5, 10, 20 μM) and measured subsequent NO overproduction. According to Khorasani et al., LPS increases the production of various inflammatory cytokines, including iNOS and COX-2, in human dental pulp cells [30]. Lechner and Tanaka demonstrated that anti-inflammatory compounds may result from an inhibitory effect on iNOS, inflammation-related cytokines, and LPS-induced NO production [31,32]. Therefore, this study compared cells treated with only LPS and not treated with CBC, and the untreated control group exhibited significantly lower NO production (50% in 20 μM). Conversely, NO was found to be overproduced during LPS treatment. Further, NO overproduction had decreased in a concentration-dependent manner following CBC treatment at different concentrations on LPS-induced RAW 264.7 cells. At a CBC treatment concentration of 20 μM, NO production was the lowest at 49.75% (Figure 2B).

### 2.2. Effects of Cannabichromene on mRNA Expression of Inflammation-Related Genes

**Figure 3:**
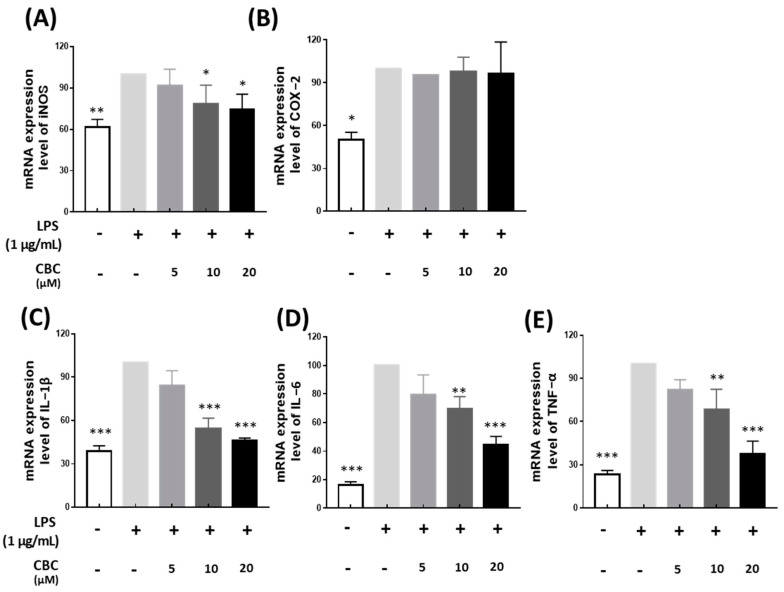
The inhibitory impact of CBC on the expression of inflammation-inducing mRNA. All samples, except the control, were subjected to LPS treatment at a concentration of 1 μg/mL. Comparative analysis was conducted for (**A**) iNOS, (**B**) COX-2, (**C**) IL-1β, (**D**) IL-6, and (**E**) TNF-α mRNA levels relative to the LPS-only experimental group. The data is presented as means ± standard deviation for triplicate experiments. *p*-values were calculated using ANOVA and Tukey’s test based on the LPS-only data, * *p* < 0.05, ** *p* < 0.01, *** *p* < 0.001.

We explored how CBC affects inflammatory cytokine production and gene transcription associated with inflammation using RT-PCR. The set of examined inflammatory genes comprised iNOS and COX-2, along with the proinflammatory cytokines IL-1β, TNF-α, and IL-6. NO produced by iNOS causes atherosclerosis, tumorigenesis, and apoptosis [33]. Moreover, it is well-established that the dysregulation in the synthesis of the measured cytokines is a contributing factor to conditions such as systemic inflammatory response syndrome [34]. The inflammatory genes were examined to determine whether they were inhibited by CBC in LPS-treated RAW 264.7 cells. In the LPS-treated group, the mRNA of all inflammatory genes that were measured in the non-LPS-treated control group were not expressed, except for COX-2; the expression of all genes was suppressed at CBC concentrations of 5, 10, and 20 μM (Figure 3). The iNOS expression rate was suppressed by 74.12% compared with the LPS-treated group at the highest concentration of CBC (20 μM); the expression of inflammatory cytokines IL-1β, TNF-α, and IL-6 was also suppressed by 46.22, 44.33, and 37.59%, respectively, at the highest treatment concentration (Figure 3A, C–E). Therefore, CBC suppressed the mRNA expression of iNOS, IL-1β, IL-6, and TNF-α, except for COX-2, in a concentration-dependent manner in RAW 264.7 cells (Figure 3). These results indicate that CBC may act as an anti-inflammatory agent.

### 2.3. Investigation of Protein Expression of iNOS, COX-2 and Inflammatory Cytokines According to Cannabichromene Treatment in Lipopolysaccharide-Treated RAW 246.7 Cells

**Figure 4:**
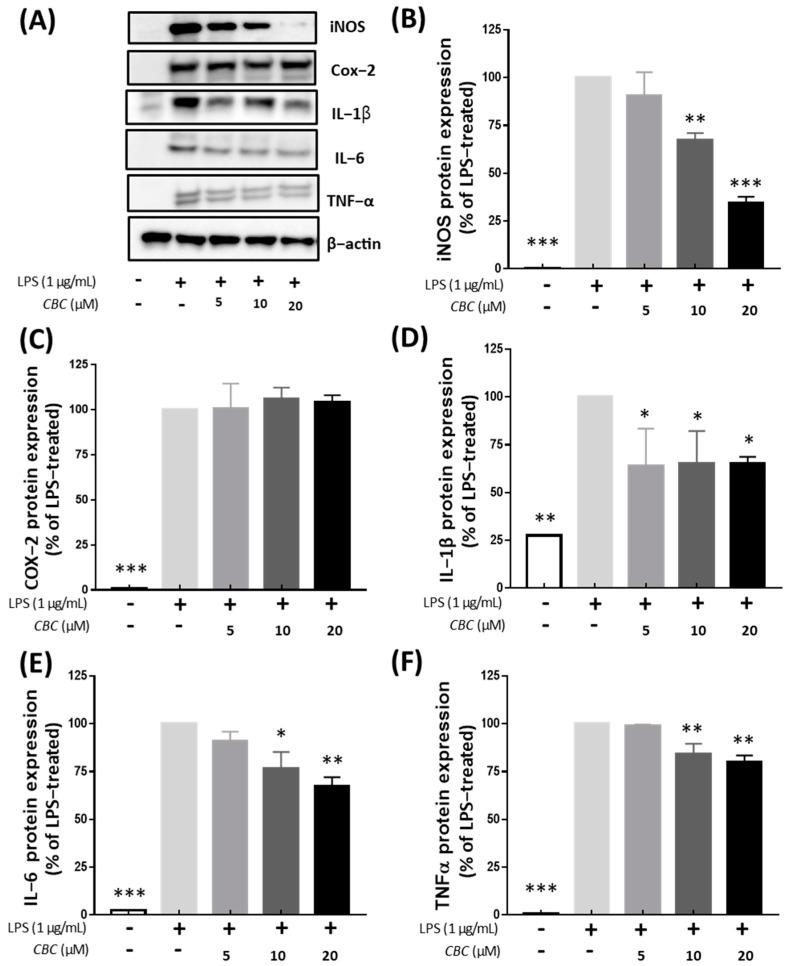
Inhibitory effect of CBC on inflammation-inducing proteins. (**A**) shows Western blot bands for individual proteins, while (**B**) compares the protein expression levels of iNOS, (**C**) COX-2, (**D**) IL-1β, (**E**) IL-6, and (**F**) TnF-α. *p*-values were calculated from the only LPS-treated group utilizing ANOVA followed by Tukey’s test; * *p* < 0.05, ** *p* < 0.01, *** *p* < 0.001.

We analyzed the impact of CBC on the synthesis of inflammatory cytokines and inflammation-related proteins. All inflammatory proteins were not expressed in the control group without LPS treatment. Except for the COX-2 protein, the expression of all inflammatory proteins was decreased following exposure to CBC concentrations of 5, 10, and 20 μM. Unlike iNOS, which mediates nitrate production, COX-2 is responsible for the synthesis of prostaglandins at the inflammatory site during inflammation, participates in various inflammatory reactions, and is involved in the development of pulpitis [35,36,37]. However, unlike iNOS, CBC did not significantly inhibit COX-2 expression, which may be the result of inadequate therapeutic doses or other mechanisms of action of CBC. In the case of iNOS, the protein expression rate was 34.40% at the highest treatment concentration of 20 μM (Figure 4B). In addition, the protein expression of inflammatory cytokines IL-1β, TNF-α, and IL-6 were reduced by 66.37, 67.21, and 79.85%, respectively, compared with the control group not treated with CBC (Figure 4D–F). These findings align with the decreasing trend observed in mRNA levels, indicating that CBC inhibits LPS induction.

### 2.4. Inflammation Regulation of Cannabichromene in the MAPK Pathway

**Figure 5:**
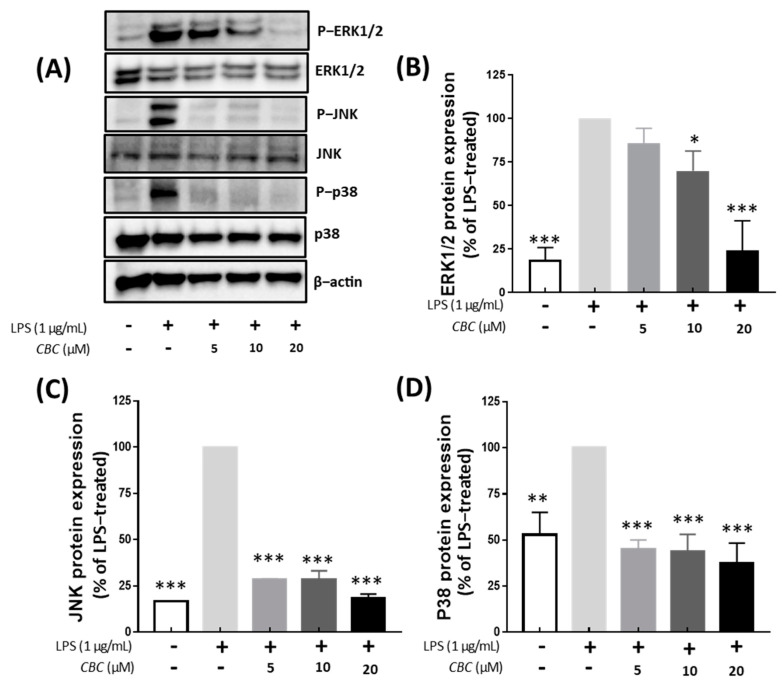
Effects of CBC on the inhibition of signaling pathways activating MAPK. (**A**) depicts the Western blot illustrating the expression of each protein. (**B**) compares the protein expression levels of ERK1/2, (**C**) JNK, and (**D**) P38. *p*-values were calculated from the only LPS-treated group utilizing ANOVA followed by Tukey’s test; * *p* < 0.05, ** *p* < 0.01, *** *p* < 0.001.

To confirm the effects of CBC on the MAPK pathway, the expression of JNK, ERK, and P38 proteins was examined. The inhibition of ERK1/2, JNK, and P38 signaling pathways is known to attenuate the inflammatory response to LPS [38]. Compared with the LPS-treated group, no phosphorylation was observed in the untreated controls. In addition, phosphorylation of JNK, ERK1/2, and P38 was inhibited at all concentrations of CBC (5, 10, and 20 μM). MAPK inhibition by CBC was the strongest with 18.47% for JNK, followed by 24.13% for ERK, and 37.58% for P38 at the highest treatment concentration of 20 μM. We observed that CBC suppressed physiological responses and hindered inflammatory factors through the inhibition of MAPK phosphorylation (Figure 5). In the aforementioned MAPK pathways, all activities were inhibited when CBC was added, suggesting that CBC can improve the inflammatory response.

### 2.5. Cannabichromene Inhibition of NF-κB Phosphorylation

**Figure 6:**
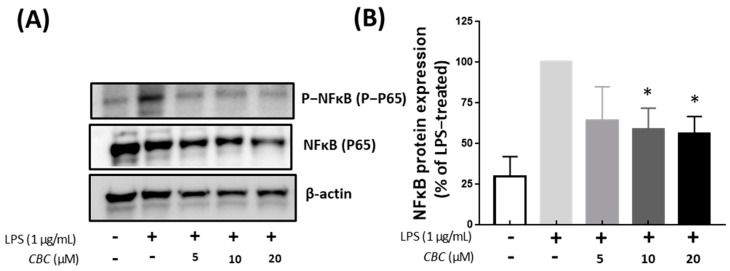
The effect of CBC on NF-κB inhibition. (**A**) is a Western blot showing protein expression, whereas (**B**) presents NF-κB protein expression levels relative to the LPS-only experimental group. *p*-values were calculated from the only LPS-treated group utilizing ANOVA followed by Tukey’s test; * *p* < 0.05.

Because NF-κB plays a principal role in the expression of cytokines and induced enzymes, we investigated the effect of CBC on the NF-κB pathway [32]. NF-κB is also activated by LPS and induces pulp inflammation [39,40]. Compared with the LPS-treated group, NF-κB phosphorylation was not confirmed in the untreated controls, whereas NF-κB phosphorylation was suppressed by CBC treatment compared with the LPS-only experimental group. Thus, phosphorylation was inhibited at 55.86% at the highest concentration of CBC treatment at 20 μM (Figure 6B). The regulation of the MAPK pathway by CB1 and CB2 receptors [41,42,43] and their involvement in apoptosis through NF-κB [44] have been well-documented. Additionally, within the spectrum of TRPV receptors—which serve as prominent target receptors for CBC—TRPV1 and TRPV4 have been recognized for their regulatory roles in the MAPK and NF-κB pathways [45,46]. In our study, the application of 20 µM CBC resulted in a reduction in the increased protein expression induced by LPS for p44/42 MAPK (ERK1/2), JNK, and P38, concurrently inhibiting NF-κB phosphorylation. This observation unequivocally confirms that CBC functions as an inhibitor of the MAPK and NF-κB pathways (Figure 5 and Figure 6). Thus, it is believed to constitute a central mechanism driving anti-inflammatory effects within macrophages [47,48].

### 2.6. Inhibitory Effects of Cannabichromene on λ-Carrageenan-Induced Mouse Model

Screening of anti-inflammatory activity by inducing inflammation in mouse paws with λ-carrageenan is a well-known experimental model of acute inflammation [49,50]. Furthermore, according to Burayk et al. [51], experiments involving carrageenan treatment in mice led to an increase in inflammatory factors, and it was confirmed that the expression of these inflammatory factors could be reduced by oral administration of anti-inflammatory compounds. When λ-carrageenan is inoculated into the soles of mice, prostaglandins are released by histamine, serotonin, bradykinin, and COX-2 within approximately 1 h, causing inflammation and neutrophil infiltration [51]. In addition, neutrophil-derived NO and pro-inflammatory cytokine release induce acute inflammation [52].

**Figure 7:**
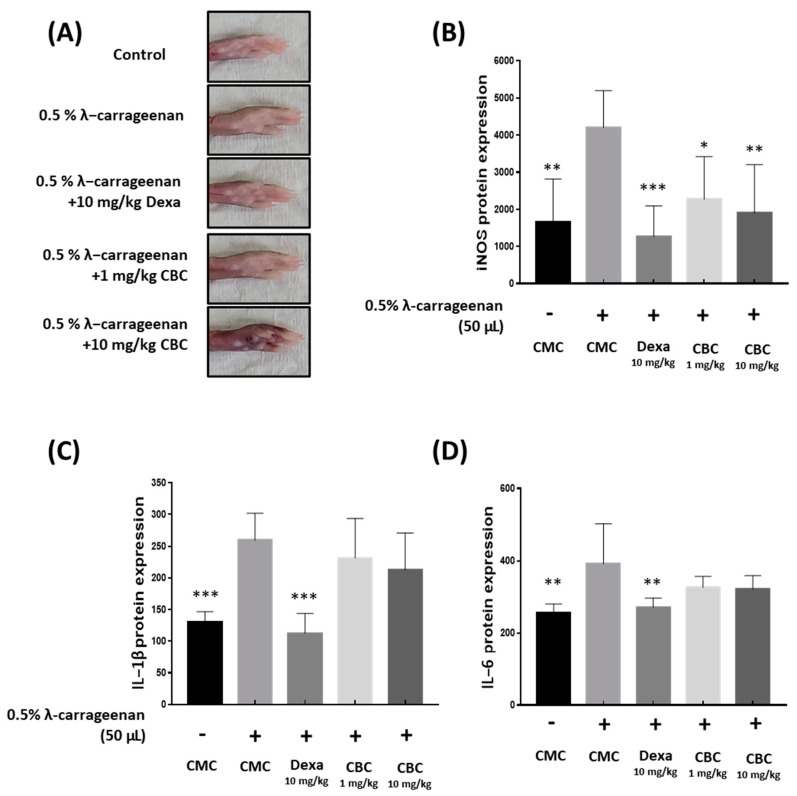
The impact of CBC on iNOS, IL-1β, and IL-6 reduction in cytokines induced by λ-carrageenan in mice. (**A**) Measuring mouse paw swelling in each experimental group after 4 h of λ-carrageenan treatment. Evaluation of the production of inflammatory factors, containing (**B**) iNOS, (**C**) IL-1β, and (**D**) IL-6, was performed using ELISA. *p*-values were calculated from the experimental data of the only LPS-treated group utilizing ANOVA followed by Tukey’s test; * *p* < 0.05, ** *p* < 0.01, *** *p* < 0.001. CMC, 0.5% carboxymethylcellulose.

To confirm the reduction in inflammatory factors following oral administration of CBC, we inoculated 0.5% carrageenan into the paws of mice [11,12] (Figure 7A). The edematous tissues were removed, and proteins were extracted to analyze the inflammatory factors using enzyme-linked immunosorbent assay (ELISA). The iNOS levels, compared with those in mice only injected with 0.5% carrageenan, decreased by 55% following oral administration of CBC 10 mg/kg. In contrast, IL-1β and IL-6 levels did not show any significant changes after oral administration of CBC (Figure 7B–D). These results indicate that CBC administration reduced IL-1β and IL-6 levels in vivo and inhibited iNOS inflammatory responses in mice.

## 3. Materials and Methods

### 3.1. Plant Materials

For this study, we used *Cannabis sativa* L. cv. Pink pepper (GenBank No: GCA_029168945.11). It was developed by Lim in 2022 and cultivated in Chuncheon, Republic of Korea, by the Chuncheon Bio-industry Foundation (CBF) (coordinates: 37°53′33″ North; 127°44′38″ East) [21,22]. The hemp was subjected to a drying process in a hot-air drying device (Daedong, KAPD-195D, Seoul, Republic of Korea) for 50.0 h at 45.0 °C. Afterward, it was further ground through an 80-mesh size grinding machine (Daesung, Artlon, DA280-S, Republic of Korea) and kept at 23 °C with 14% humidity in a thermo-hygrostat (Daihan, DH.DeADDBG1K, Wonju, Republic of Korea).

### 3.2. Supercritical Fluid Extraction Procedure for Cannabichromene Extract

A 1000 g sample of dried hemp was introduced into a 10 L supercritical fluid extractor (SFE) vessel. The vessel was sealed to prevent gas escape, and the SFE was operated for 110 min, adhering to the following parameters: CO_2_ flow rate of 500.0 g/min, sampler temperature set at 54.0 °C, separator temperature maintained at 30.0 °C, and pressure regulated to 7000 psi (Phos-enthech, SFE-10L, Daejeon, Republic of Korea). After the extraction process was completed, the pull out was incubated at 25 °C for about 2 h until the carbon dioxide was removed. Subsequently, the volume of the produced pull out was measured.

### 3.3. Cannabichromene Purification

To obtain high-purified CBC by SFE-extracted material, we added 99% ethanol in a tenfold quantity compared to the extract. This mixture was then stored in a deep freezer (Thermo Fisher, 907, Waltham, MA, USA) at −72.0 °C for 20 h. To remove the wax layers, the mixture underwent filtration using filter paper (Whatman WF2-0900, Maidstone, UK) and a vacuum filter. Subsequently, the ethanol in the evaporative process was applied to the filtered extract using a rotavapor (R-220SE, Büchi, Flawil, Switzerland) under conditions of 70 bar pressure and 45 °C for 60 min. The resulting extract was subsequently subjected to MPLC (Bio-tage, SIO-1EV, Charlotte, NC, USA). The separation of CBC was carried out using an instrument equipped with Sfär C18 D (Biotarge, FSUD-0401-0120, Hengoed, UK), while a mixture of 70% ethanol and distilled water flowed through the column at a rate of 50 mL/min. After the solvents were removed from the separated CBC using a vacuum concentrator, a product with 99% purity was obtained.

### 3.4. Cell Culture

RAW 264.7 cells were purchased from the American Type Culture Collection (ATCC, Rockville, MD, USA). These cells were grown in DMEM (Dulbecco’s Modified Eagle’s medium) supplemented with 10% fatal bovine serum (FBS) and 1% penicillin at 37 °C with 5% CO_2_.

### 3.5. Cell Viability and Production of Nitric Oxide

To assess cell viability, the RAW 264.7 cells were incubated at a density of 1 × 10^4^ cells/well for 18 h in a 96-well plate, treated with increasing concentrations of CBC 5, 10, and 20 μM, respectively, for 24 h. Cell viability was confirmed using a Cell Counting Kit-8 assay (CCK-8, #SE814 Dojindo Molecular Technologies, Rockville, MD, USA) according to the manufacturer’s protocol.

RAW 264.7 cells were incubated for 24 h in a 24 well-plate at 2 × 10^5^ cells/well and were treated with lipopolysaccharide (LPS, Sigma-Aldrich, St Louis, MO, USA) at a concentration of 1.0 μg/mL. After 2 h of LPS treatment, cells were treated with 5, 10, and 20 μM CBC for 18 h, respectively. NO production was evaluated by nitrite measurement using a Nitric Oxide Plus Detection kit (iNtRON, Seongnam, Republic of Korea).

### 3.6. RT-qPCR

RAW 264.7 cells, plated at a density of 2.5 × 10^5^ cells per well in a 6-well plate, were incubated for 24 h and subsequently treated with LPS at 1.0 μg/mL. After 2 h of LPS treatment, the RAW 264.7 cells were treated (2.5 × 10^5^ cells/well) with 5, 10, 20 μM CBC, and the mRNA was isolated using an RNA isolation kit (Qiagen, Inc., Valencia, CA, USA). RNA purity was confirmed using a NanoDrop analyzer (Thermo Fisher Scientific, Madison, WI, USA). The RNA was transcribed into cDNA using a reverse transcription master mix (Elpis-Biotech, Dae-jeon, Republic of Korea), and the relative expression levels were confirmed using a LightCycler480 Instrument II (Roche, Mannheim, Germany). The oligonucleotide primers were as follows: iNOS, NM_010927.4 (F: AATGGCAACATCAGGTCGGCCATCACT; R: GCTGTGTGTCACAGAAGTCTCGAACTC), Cox-2, NM_011198.5 (F: GGAGAGACTATCAAGATAGT; R: ATGGTCAGTAGACTTTTACA), IL-1β, NM_008361.4 (F: TGCAGAGTTCCCCAACTGGTACATC; R: GTGCTGCCTAATGTCCCCTTGAATC), IL-6, DQ788722.1 (F: GAGGA-TACCACTCCCAACAGACC; R: AAGTGCATCATCGTTGTTCATACA), TNF-α, LN874395.1 (F: ATGAGCACAGAAAGCATGATC; R: TACAGGCTTGTCACTCGAATT), GAPDH, GU214026.1 (F: GTATGACTCCACTCACGGCAAA; R: GGTCTCGCTCCTGGAAGATG).

### 3.7. Cell Lysate Preparation, Isolation of Nuclear Extracts, and Subsequent Western Blotting Analysis

RAW 264.7 cells were incubated in a 6-well plate at a density of 2.5 × 10^5^ cells per well, and subjected to LPS treatment at 1.0 μg/mL for 2 h. Following the LPS treatment, the cells were exposed to 5, 10, and 20 μM CBC for an additional 18 h. Protein lysates were obtained using PRO-PREP™ (iNtRON Biotechnology, Seongnam, Republic of Korea). The protein concentration was determined using the Pierce™ BCA Protein Assay (Thermo Fisher Scientific, Rockford, IL, USA). Protein analysis was performed through Western blotting, which involved SDS-PAGE and antigen-antibody interactions. The primary antibodies were acquired from Cell Signaling Technology (Danvers, MA, USA). Antibody signals were detected using Supersignal West Pico (Pierce, Rockford, IL, USA) and were verified using the LAS-4000 imaging system (Fujifilm, Tokyo, Japan).

### 3.8. Mouse Experiment

Male C57BL/6J mice at 6–7 weeks of age were bred with ad libitum consumption of food and water under standardized conditions of temperature (24 ± 2 °C), humidity (55% ± 5%), and light (12 h light/12 h dark cycle). Acute inflammation was induced in the right hind paw using 50 μL of 0.5% carrageenan (Sigma-Aldrich, St. Louis, MO, USA), and sterile saline was injected into the control group. For comparison with CBC, 10 mg/kg of dexamethasone was administered and utilized as a positive control. CBC was orally administered using a sonde 3 days before and on the day of injection of 0.5% carrageenan. Four hours after the 0.5% carrageenan injection, paw edema was measured using calipers, and hind paws were excised, flash-frozen in liquid nitrogen, and ground using a mortar and pestle. Proteins were extracted from the tissue samples and ground in a mortar using PRO-PREP™ (iNtRON Biotechnology, Seongnam, Republic of Korea). The levels of iNOS, IL-1β, and IL-6 were analyzed using a commercial ELISA kit. All experiments were conducted in accordance with the guidelines of the Institutional Animal Care and Use Committee (IACUC) of the CBF granted approval under the number CBF-IACUC-2023-015.

### 3.9. Data Analysis

All experiments were independently conducted and replicated three times or more. The findings are expressed as mean ± standard deviation. Graphs illustrating the experimental data were produced using GraphPad, v.7.0.1. Statistical analysis for each experimental dataset was conducted using one-way ANOVA followed by Tukey’s multiple comparisons test, with significance levels set at 5%, 1%, and 0.1%.

## 4. Conclusions

**Figure 8:**
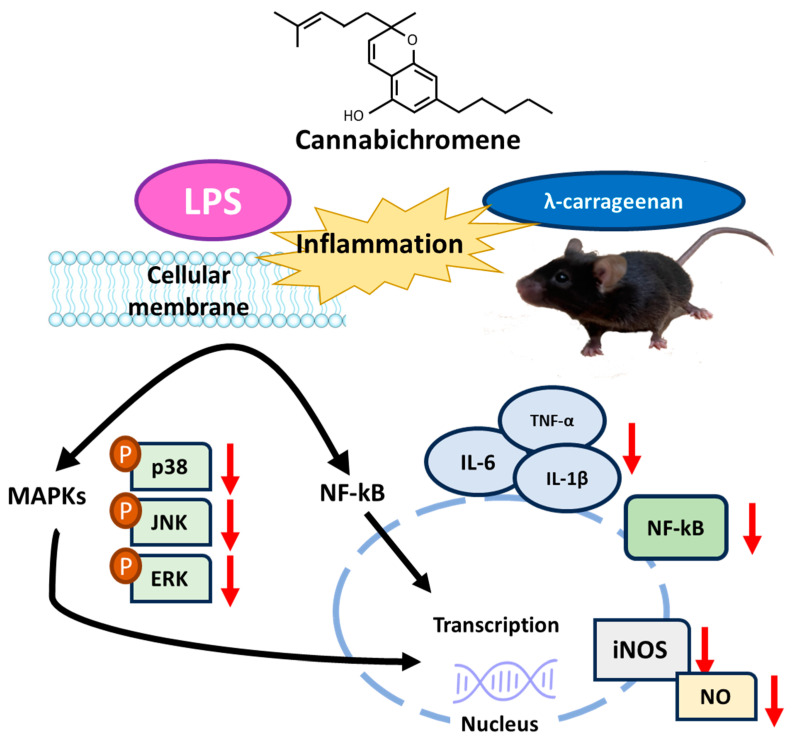
Summary of mechanisms of suppression of inflammatory signaling by CBC.

CBC exerts various physiological effects, and our study has demonstrated its potent anti-inflammatory effects. The inhibitory effects of CBC on inflammatory markers found in this study through in vitro and in vivo experiments induced by LPS and λ-carrageenan can serve as evidence to support the utilization of CBC to reduce inflammation. Although overexpression of inflammatory mediators leads to nitric oxide production, we found that the levels were reduced following CBC treatment. The outcomes of our study highlight the potential of CBC as an effective treatment for a spectrum of diseases linked to both chronic and acute inflammatory reactions (Figure 8). Thus, CBC extracted from the newly developed Pink Pepper variety holds substantial industrial, pharmacological, and physiological value. Furthermore, the anti-inflammatory effects of CBC derived from *Cannabis sativa* L. in this study provide scientific support for the medicinal use of *Cannabis sativa* L.

## Data Availability

Data are contained within the article. Materials of the CBC isolated from ‘Pink Pepper’ provide from the corresponding authors.
